# Molecular Detection of *Nosema* spp. in Three Eco Regions of Slovakia

**DOI:** 10.3390/cimb45060306

**Published:** 2023-06-01

**Authors:** Beáta Hurná, Monika Sučik, Martin Staroň, Štefan Tutka, Zuzana Maková, Richard Galajda, Alexandra Valenčáková

**Affiliations:** 1 Department of Biology and Physiology, University of Veterinary Medicine and Pharmacy in Košice, Komenského 73, 041 81 Košice, Slovakia; monika.sucik@uvlf.sk (M.S.); zuzana.makova@uvlf.sk (Z.M.); richard.galajda@uvlf.sk (R.G.); alexandra.valencakova@uvlf.sk (A.V.); 2 VÚŽV Nitra-Institute of Apiculture Liptovský Hrádok, Gašperíkova 599, 033 80 Liptovský Hrádok, Slovakia; martin.staron@nppc.sk (M.S.);

**Keywords:** *Nosema apis*, *Nosema ceranae*, multiplex PCR, diagnostics, Slovakia

## Abstract

Microsporidia are unicellular obligate intracellular parasitic fungi that infect a wide range of vertebrates and invertebrates. There are two known species of microsporidia infecting honey bees in Slovakia- first *Nosema apis* and also *Nosema ceranae.* Our aim was to examine samples of honey bees collected from bee queen breeders in three ecoregions of the Slovak Republic in 2021 and 2022. First, microscopic diagnostics were used, and then randomly selected samples were examined using molecular methods. There were 4018 samples examined using microscopic diagnostics and the positivity was demonstrated in 922 samples. From the microscopically diagnosed positive samples, 507 samples were randomly selected, and using molecular methods, the positivity was proved in 488 samples. After sequencing the positive PCR products and comparing the sequences (BLAST) with the sequences stored in the gene bank, the *Nosema ceranae* species was detected in all positive samples.

## 1. Introduction

Microsporidia are unicellular [1] obligate intracellular parasites [2], parasitic fungi [3] that infect a wide range of vertebrates and invertebrates, including humans. They were discovered more than 160 years ago and include around 1600 species [4].

These parasitic fungi are eukaryotes without typical features of eukaryotes such as mitochondria, peroxisomes, and Golgi apparatus. Outside the body of their host, they exist in the form of highly resistant chitin-coated infectious spores, ranging in size from 1 to 40 μm, and may occur in water or soil [1,2]. They can be viable in the environment for over a year [5]. There are three known species of microsporidia infecting honey bees: *Nosema apis*, *Nosema ceranae,* and *Nosema neumanni*.

*Nosema apis* has been known to science for more than 100 years [2], since 1909, in the honey bee- *Apis mellifera*. In 1996, *Nosema ceranae* was first identified in the Asian honey bee, *Apis cerana*, and in 2017, *Nosema neumanni* was discovered as a new species of this parasite, which is found, so far, only in Uganda [6].

In local geographical conditions, *Nosema apis* was the first to occur, *Nosema ceranae* occurred later [7], often appearing as a co-infection. Until 2008, *Nosema apis* was the only diagnosed species of the microsporidian parasite in Slovak honey bees causing diarrhea [8]. This disease is often associated with CCD—colony collapse disorder [9].

Nosematosis causes physiological and behavioral changes in honey bees [3]. Clinical signs begin to appear only after the spores multiply in the epithelium of the digestive system [10]. During *Nosema apis* infection, one may easily notice the bee’s fecal material on the honey frames and in the area of the hive, and crawling honey bees around the hive [7].

The honey bees infected with *Nosema ceranae* do not show clinical signs of the disease [2], despite a significantly higher spore load in the digestive system than in the case of *Nosema apis* infection.

The infection occurs after ingestion of water or food containing mature spores, most often via the fecal–oral route or during tropholaxy. The spores are located in the pollen, honey, and royal jelly that is fed to the larvae. Transmission of infection is also possible through contaminated beeswax combs in the hive [2,3].

Transmission might be horizontal-from the drone’s sperm to the bee queens during mating [11]—but infection is also possible during the artificial insemination of the bee queens. The infected are the worker bees and the larvae that are fed by the bees. Vertical transmission from a bee queen to an egg has not yet been confirmed [12].

The life cycle of *Nosema* spp. begins after the infective spore has been ingested and takes place in the digestive tract of bees. It consists of a period of merogony, when meronts are formed, which is the period of spore reproduction in the digestive tract. This is followed by a period of sporogony of mature spores, when sporonts are formed, which multiply rapidly in the cell until they cause lysis. Infected cells burst and release mature spores into the lumen of the digestive tract [1,11].

Spores present in the lumen of the digestive tract germinate and release polar filaments from the spores, which attach to and infect new cells of the digestive tract epithelium. Some of the spores are expelled into the environment and some remain in the gut [5].

Bees affected by *Nosema* spp. infection suffer from malabsorption and excrete significant amounts of undigested sugar in their feces, which is an attractant and often a source of infection for other bees [13]. In addition to the digestive tract, *Nosema* spp. spores can also reach the hypopharyngeal glands via the hemolymph, which are required for the production of royal jelly that is fed to the young bees and queen, thus slowing down the development of the whole colony and weakening it. Nosema uses substances and energy from the cells of the host’s digestive tract to promote their own reproduction [14].

Bees defend against infection by programming cell death through apoptosis. It is a defense mechanism that invertebrates use against viruses and other intracellular pathogens [15]. Nosema causes the inhibition of apoptosis and regulates it to its advantage. The host can tolerate the pathogen and repair the damage it causes in the organism by proliferating new cells in the intestinal epithelium [16].

As a result, the symbiotic microflora of the bee’s digestive tract is altered, disrupting the homeostasis of the organism, enzyme production, and food absorption in the midgut. With the destruction of the digestive tract epithelium, cell necrosis occurs, leading to bee mortality [14].

The only approved treatment to combat Nosema infection is the antibiotic FUMAGILIN-B, which has been commercially unavailable to beekeepers since 2018 [17]. The use of antibiotics for the treatment of honey bee colonies in the countries of the European Union is prohibited with zero tolerance for honey. There is a potential risk of pathogen resistance and contamination of the hive environment with antibiotics [18]. Probiotics are used as an alternative method in the fight against the disease. This treatment has a beneficial effect on the health and longevity of honey bees, improves intestinal microbiota and homeostasis, eliminates pathogens from the digestive system, stimulates the immune system, and aids in food digestion [19]. As a preventive measure, it is necessary to remove the contaminated parts of the hive. This measure will help remove the spores and thereby suppress the disease [17].

Many methodologies have been used in the diagnosis and study of infections caused by *Nosema* spp. Light microscopy is the primary method for detecting *Nosema* spp. [13]. In addition to detecting the spores of the pathogen, light microscopy also helps us to evaluate the infection rate of infected bees [5].

Diagnostics are made by laboratory testing and a demonstration of the pathogen from recently dead bees, which show clinical signs or are suspected of having the disease, by microscopy [7]. The principle of this method is the observation of spores with a light microscope at 400× magnification in a drop of crushed bee abdomens mixed with a physiological solution [13].

Nosema spores have a regular shape, size, and so-called refraction of light under the microscope. *Nosema apis* may be distinguished from *Nosema ceranae* by the size of the spores. The spores of *Nosema apis* have a shape between oval to barrel; they are from 4.9 to 6.9 μm long and 2.7 to 3.9 μm wide. *Nosema ceranae* has smaller, cylindrical spores with a pointed end measuring from 3.9 to 5.4 μm in length and 2.0 to 2.9 μm in width [20].

To quantify spore counts, a hemocytometer count is used, where we count the number of spores in all squares of the hemocytometer and, after plugging into the formula, calculate the number of spores per bee [21]. To better detect spores, they can be stained by Giemsa staining with a commercially available dye; this method is used to distinguish *Nosema* spp. spores from other microbes in the sample [22], or we can use toluidine staining [21]. Using fluorescence microscopy, we can distinguish live spores from dead spores of *Nosema cerenae* in addition to detecting infection.

Among microscopic diagnostics, transmission electron microscopy—TEM—is the gold standard in identifying microspores, which helps to distinguish *Nosema* spp. based on the observation of the number of their polar filaments in the sporoplasm.

The histopathological method is a very sensitive and specific method for the detection of nosematosis and has the advantage of being able to provide a diagnosis in colonies without signs of disease [23].

Microscopic quantification and detection of Nosema spores is a reliable method of detection. However, differential diagnosis is not performed microscopically, except by TEM, which is time-consuming and also expensive [13]. Therefore, the polymerase chain reaction method was developed as a tool to diagnose the species [5]. PCR is a very sensitive method that enables the detection of the parasite even at very low levels of infection and may identify all stages of the microsporidia’s life cycle [21]. In particular, multiplex PCR, PCR-RFLP, and qPCR are used [24]. The first step of the reaction is the isolation of DNA from the samples, following a tissue protocol using commercially available kits [5]. This extracted DNA is the template for the preparation of the PCR mix to which special commercially available primers for the detection of *Nosema* spp. are added [25]. The use of primers designed based on the 16S rRNA minor subunit gene is recommended as the most reliable method. DNA amplification is performed on a thermocycler, followed by the visualization of PCR products on agarose gels. The resulting PCR reaction products can then be sent for sequencing, where the exact sequence is harvested, and the result is compared to the sequences in the Gene Bank [13,21].

In addition to the aforementioned types of PCR, diagnosis using the LAMP method is also possible, during which both *Nosema* spp. can be detected. This method is based on the isothermal amplification of DNA fragments and the authors report that it is highly specific and even more sensitive than PCR alone [26,27].

In field settings, the immunodiffusion serological method can be used, which is mainly suitable for the detection of *Nosema cerana* [28].

All methodologies for the diagnosis of *Nosema* spp. are described in the BEEBOOK practical manual of standard methods for the honey bee [21].

The aim of this pilot study is to investigate the incidence of *Nosema* spp. in three ecoregions of Slovakia in 2021 and 2022 by comparing our results with those of other countries that have performed species diagnostics in their territory and monitored the prevalence of *Nosema* spp., and to compare the accuracy of the microscopic diagnoses with molecular methods.

## 2. Materials and Methods

### 2.1. Sampling

Samples of dead honey bee workers for the detection of Nosemosis and Acarapidosis were collected by the bee queen breeders in the breeding farms from bottom hive debris. Sampling of dead honey bee workers for the examination was performed at the beginning of the year in January and February. The beekeepers sent the samples, whose number is specified to be 30–50 bodies per bee colony, to the Institute of Apiculture Liptovský Hrádok.

In 2021, 39 bee queen breeders were registered in Slovakia and a total of 2028 samples were sent for examination. In 2022, 32 bee queen breeders were registered and a total of 1990 samples were sent for testing. The number of keepers from each part of the country is given in Table 1.

### 2.2. Sample Preparation for Microscopic Examination of Nosema *spp.*

The abdomens of 50 dead winter honey bees were cut from their bodies. Then, the abdomens were crushed using a mortar and pestle in distilled water.

After the bee abdomens were crushed with a mortar and pestle, the solid part was separated from the liquid part. A drop of the liquid part was placed on the glass slide and covered with a coverslip. The preparation was viewed under a microscope with phase contrast 400× magnification, as it helps distinguish Nosema spores from yeast or other particles. The Nosema spores were observed under the microscope from several fields of view, and then the results of the number of spores were averaged and evaluated. In this case, 0 spores viewed were evaluated as negative samples, 1–19 spores in the field of view were determined as + weak Nosema infection, 20–100 spores in the field of view were identified as ++ moderate Nosema infection, and above 100 spores in the field of view were evaluated as +++ strong Nosema infection. The liquid part of the samples, which was evaluated under the microscope as positive for *Nosema* spp., was labeled in test tubes, frozen, and sent for molecular diagnosis and detection of *Nosema* spp. to the University of Veterinary Medicine and Pharmacy in Košice.

### 2.3. PCR-Based Diagnostics of Nosema *spp.*

The frozen samples of the liquid component of honey bee abdomens were thawed at room temperature. In order to isolate DNA from the samples, DNA-sorb-AM (AmpliSens, Moscow, Russia) kit for the extraction and purification of nucleic acids from tissues was used according to manufacturer’s instructions. Then, 0.5 mm and 1.0 mm zirconium beads were added to each sample in order to break hard coat of Nosema spores; PRECELLYS 24 (Bertin, MD, USA) tissue homogenizer was used, and the device was set to 6500 rpm and 2 × 45 s.

### 2.4. Reaction Mixture Preparation

A duplex PCR protocol by [29] was used for the preparation of the reaction mixture, which was modified as follows: 2.5 µL buffer, 2 µL MgCl_2_, 0.5 µL dNTPs, 0.3 µL DNA polymerase (FIREPol DNA Polymerase produced by Solis BioDyne, Tarfu, Estonia), and was used for a single sample. Then, we proceeded as instructed in their protocol. In order to diagnose species of *Nosema* spp., two 30 picomole primers were added to the reaction mixture, 0.6 µL to each (*Nosema apis* for the amplification of 321 bp) APIS FOR-5′-GGGGCCATGTCTTTGACGTACTATGTA-3′ and 321 APIS REV 5′-GGGGGGCGTTTAAAATGTGAAACAACTATG-3′ and (*Nosema ceranae* for the amplification of 218 bp) MITOC FOR-5′CGGCGACGATGTGATATGAAAATATTAA-3′ and 218 MITOC REV 5′-CCCGGTCATTCTCAAACAAAAAAACCG-3. 2.5 µL of DNA template was added to the mixture, and PCR water was filled to final volume of 25 µL.

PCR was performed using a VWR RISTRETTO Personal Thermocycler according to the protocol of Ostroverkhová et al., 2020. ELFO was performed using 1.5% agarose gel after PCR reaction had been completed. After assessing the results using a UV Transilluminator, the DNA concentration was measured using the NanoDrop, and the PCR products were sent for sequencing. The obtained sequences were compared with the sequences stored in the gene bank using BLAST program. https://blast.ncbi.nlm.nih.gov/Blast.cgi (accessed on 20 November 2022).

### 2.5. Statistical Analysis

We performed statistical analyses of our results. To obtain a representative value, we calculated the mean, followed by the median, minimum value, maximum value, and coefficient of determination. To see if there is a linear dependence between our numbers of positive samples in both years and also the numbers of negative samples, we calculated the correlation coefficient between the positive and negative samples using the coefficient of determination-R^2^

Statistical analyses were performed using the Microsoft Excel software package. To calculate the correlation from the calculated value of determination, we used the formula for calculating the correlation coefficient: R = √(R^2^).

## 3. Results

### 3.1. PCR Diagnostics of Nosema *spp.*

**Figure 1:**
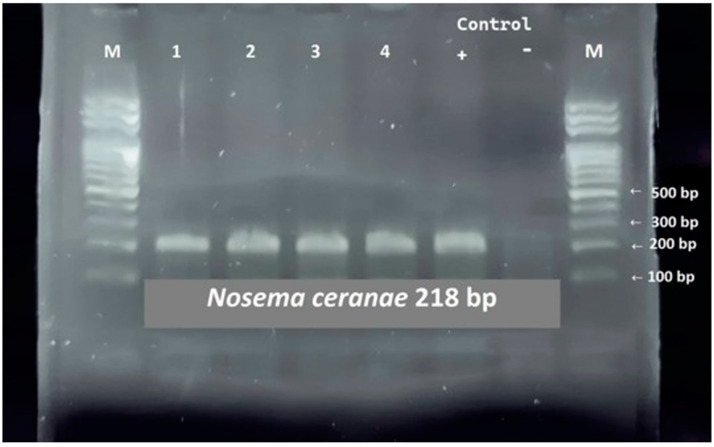
Agarose gel showing PCR products amplified from *Nosema apis* and *Nosema ceranae*; the presence of *Nosema ceranae* was confirmed in all samples shown in lanes 1–4, lane 5: Positive control, lane 6: Negative control, lane M: Ladder 100 bp.

All samples, which underwent DNA isolation and subsequent PCR amplification using primers for *Nosema apis* (amplicon 321 bp) and *Nosema ceranae* (amplicon 218 bp) after the application of an agarose gel and consecutive electrophoresis using UV Transilluminator, were visualized and determined as being positive for *Nosema ceranae* (Figure 1).

### 3.2. Evaluation of the Results of Examined Samples in 2021 in Three Ecoregions of Slovakia

**Figure 2:**
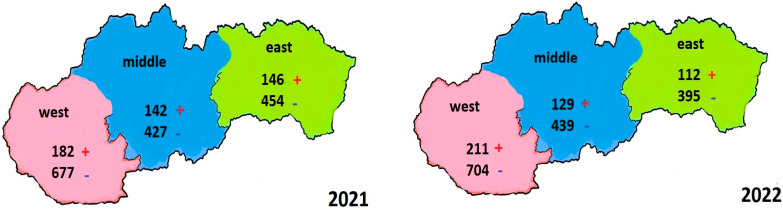
A map of Slovakia with the number of microscopically examined samples and their redistribution into the three ecoregions in 2021 and 2022.

In 2021, 2028 samples of dead honey bees collected from bee queen breeders in Slovakia were microscopically examined. A total of 859 samples were examined from the western ecoregion—182 (21.19%) were positive and 677 (78.81%) were negative for *Nosema* spp. Out of 569 samples retrieved from the middle ecoregion, 142 (24.96%) were positive, and 427 (75.04%) were negative samples. There were 600 samples collected from the eastern ecoregion—146 (24.33%) were positive and 454 (75.67%) were negative. The total balance of samples examined by the microscopic method was 470 (23.18%) positive and 1558 (76.82%) negative samples (Figure 2).

Subsequently, out of 470 positive microscopically examined samples, 81 samples of crushed honey bee abdomens were randomly selected and examined using molecular methods in order to confirm and identify species of *Nosema* spp. in Slovak honey bee colonies.

There were 40 samples examined from the western ecoregion, whereas 34 (85%) were positive and 6 (15%) were negative samples. From the middle ecoregion, there were 19 samples in total—18 (94.74%) were positive and 1 (5.26%) sample was negative.

There were also 22 samples tested from the eastern ecoregion—21 (95.45%) were positive and 1 (4.54%) negative sample. The total balance of samples examined using molecular methods was 73 (90.12%) positive and 8 (9.88%) negative samples (Table 2).

### 3.3. Evaluation of the Results of Examined Samples in 2022 in Three Ecoregions of Slovakia

In 2022, there were 1990 microscopically examined samples of dead honey bees collected from bee queen breeders in Slovakia. In total, 915 samples were examined from the western ecoregion, while 211 (23.06%) were positive and 704 (76.94%) were negative for *Nosema* spp. Next, there were 568 samples examined from the central ecoregion—129 (22.71%) were positive and 439 (77.29%) were negative. From the eastern ecoregion, 507 samples were examined, where 112 (22.09%) were positive and 395 (77.91%) were negative. The total balance of samples examined by the microscopic method was 452 positive and 1538 negative samples (Figure 2).

Consecutively, whereas there were 452 positive microscopically examined samples in total, 426 samples of crushed honey bee abdomens were randomly selected and examined using molecular methods to confirm and specify *Nosema* spp. species in honey bee colonies in Slovakia. From the area of the western ecoregion of the country, 195 samples were examined—there were 193 (98.97%) positive samples and 2 (1.03%) negative samples. Then, there were 123 samples from the middle ecoregion—118 (95.93%) tested positive and 5 (4.07%) negative. In the case of the eastern ecoregion, 108 samples were examined—104 (96.30%) were positive and 4 (2.58%) negative samples. The total balance of samples examined by molecular methods was 415 (97.42%) positive and 11 (2.58%) negative samples (Table 2).

### 3.4. Comparison of the Number of Positive Cases in Three Slovak Ecoregions from Samples Collected in 2021 and 2022

In 2021, 470 out of 2028 samples tested positive.

In 2022, 452 out of 1990 samples tested positive.

The greatest number of collected, examined, and positive samples was retrieved from the western ecoregion of the country in both years; out of 1774 in total, 393 samples were positive, whereas there were 56 more samples examined in 2022 than in 2021, and 29 more samples were positive. In the middle ecoregion of Slovakia, 1137 samples were examined in total; 142 samples were positive in 2021, and 129 samples were positive in 2022.

**Figure 3:**
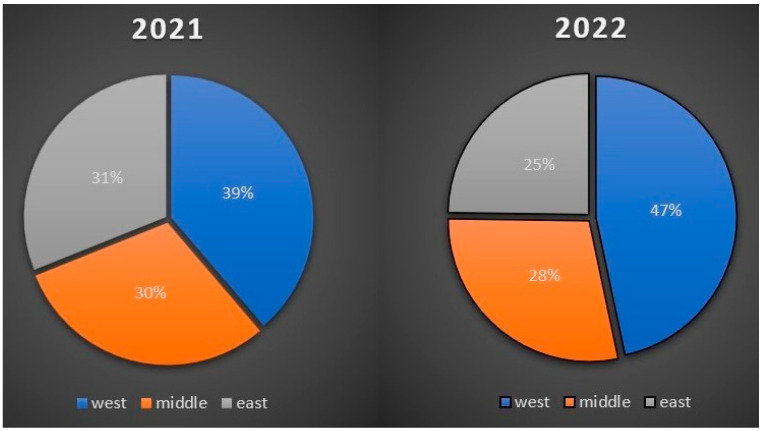
Percentage distribution of positive samples by ecoregions and years.

Taking into account the fact that the number of examined samples was almost the same, it indicates that there were 13 more positive samples in 2021. The most significant contrast between the numbers of examined samples was present in the eastern ecoregion of Slovakia. During two years, 1107 samples were examined; there were 93 more samples in 2021, and the positivity was determined in 34 more samples in comparison with 2022 (Figure 3).

### 3.5. Evaluation of Statistical Analysis of Results

The tests and analyses performed allowed us to determine the parameters described in Table 3.

The mean, the median, the minimum and maximum values, the coefficient of determination-R^2^, and the correlation coefficient-R were calculated from the R^2^ value.

The correlation coefficient for positive and negative microscopy samples for 2021, R = 0.7785, indicates to us a moderately strong dependence, and we rate the correlation coefficient for positive and negative microscopy samples for 2022, R = 0.9871, as a particularly strong correlation coefficient.

We rate the correlation coefficient for positive 2021 molecular diagnostics samples, R = 0.8950, and the correlation coefficient for positive 2022 samples, R = 0.8064, as a particularly strong correlation coefficient.

We rate the correlation coefficient for negative molecular diagnostic samples for the year 2021, R = 0.8064, and the correlation coefficient for negative samples for the year 2022, R = 0.8950, as a particularly strong correlation coefficient Table 3.

**Figure 4:**
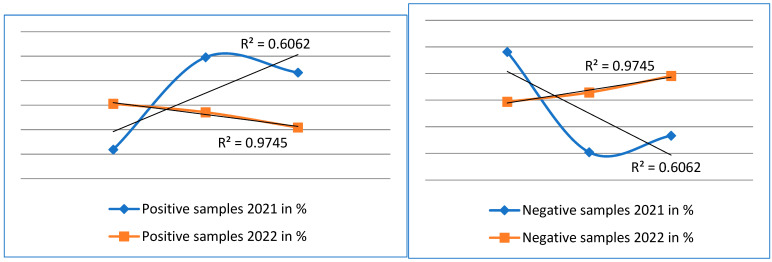
The graphs show a comparison of positive and negative samples for 2021 and 2022 examined by the microscopic method.

**Figure 5:**
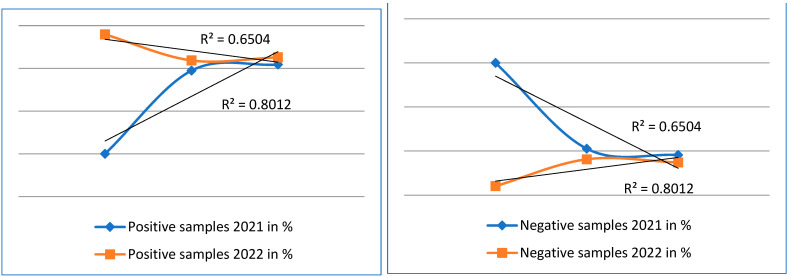
The graphs show a comparison of positive and negative samples for the years 2021 and 2022 examined by molecular methods.

From these analyses, we can conclude that there is a direct positive correlation and a direct linear dependence between the values of both the positive and negative samples for both 2021 and 2022 (Figure 4 and Figure 5).

## 4. Discussion

The present pilot study was carried out in 2021 and 2022. The aim of the study was to research the incidence of *Nosema* spp. species in Slovakia,

The identification of *Nosema* spp. in the samples was first examined by microscopic methods. Subsequently, diagnosis was performed using molecular methods that can not only detect but also determine the species of the pathogen [30]. In all 488 positive samples examined by molecular methods, *Nosema ceranae* species was detected, whose size corresponds to 218 bp [31], as presented in (Figure 1) above. Due to the high sensitivity of molecular methods, it was possible to demonstrate more accurately which samples were positive and which were negative in samples previously identified as positive using the microscopic method. The determination of false-positive samples by light microscopy over two years ranged from 2 to 8%. Statistical analyses of the results showed a strong positive linear relationship between the variables studied. We also observe a positive correlation between the number of bee queen breeders (Table 1) in 2021 and 2022 with the number of samples analyzed by microscopic methods (Table 2).

The last study of the incidence of mono and co-infections of the *Nosema apis* and *Nosema ceranae* species was conducted in Slovakia in 2009 and 2010 when *Nosema ceranae* was diagnosed in more than 90% of the samples. There was a recorded decrease in the incidence of *Nosema apis* and an increase in the incidence of *Nosema ceranae* in Slovakia [8]. Based on the latest data of the most recent (and ongoing) research that took place in 2021, *Nosema ceranae* was confirmed in all examined samples at the authors’ workplace [32].

This fact may be related to the gradual displacement of *Nosema apis* by *Nosema ceranae* from milder climatic conditions. An increasing proportion of colonies infected with *Nosema ceranae* has been documented in many regions of the world in comparison to *Nosema apis*. The increased number of co-infections suggests that *Nosema ceranae* is moving from southern mild climates to northern subtropical climate regions and the *Nosema apis* is gradually being displaced [33]. The climate could be an important factor explaining the differences in the species distribution of this parasite.

In Sweden, *Nosema apis* is still prevalent in 80% of cases and 17% are co-infections.

Co-infections prevail in Scotland, Argentina, and Australia. In Serbia and neighboring countries such as Croatia, Bosnia and Herzegovina, Montenegro, and North Macedonia, *Nosema ceranae* dominates. Furthermore, only one species—*Nosema ceranae*—may be found in Greece, Italy, eastern Azerbaijan, and Saudi Arabia [34]. *Nosema ceranae* displaces *Nosema apis* in the *Apis mellifera* population [35]. It is assumed that *Nosema ceranae* is the cause of increased colony losses registered in Europe and the United States [33]. In Spain, *Nosema* infection is even associated with reduced honey production and increased mortality. In recent years, the infection of honey bees caused by *Nosema* spp. has been reported in many European countries: Spain, France, Greece, Italy, Germany, Switzerland, Denmark, Finland, Hungary, Slovenia, and Bosna and Herzegovina.

*Nosema ceranae* was primarily present in most of the investigated honey bee colonies from honey bee societies of infected European countries. In Italy, *Nosema ceranae* is definitely widespread and has replaced *Nosema apis.*

The world trade in honey bee products and materials, and, especially, the trade in bee queens, may be a source of infection in some regions [34].

According to recent research in Bulgaria, where honey bee honey was used as a sample for DNA pathogen diagnostics, an increased prevalence of *Nosema ceranae* over *Nosema apis* was demonstrated. This points to the fact that not only the bee but also bee products such as honey can be a powerful resource for effective biomonitoring of current honey bee diseases [36].

Due to the increasing number of positive samples for *Nosema ceranae* in Europe and the world as well, which is devastating for honey bee colonies, it is necessary to continue to investigate the incidence of the species, possible mono or co-infections with *Nosema* spp., the increase in positive samples, and possible causes of the emergence and development of the infection. Last, but not least, it is important to research the possibilities of early diagnosis of *Nosema ceranae* infection and the prospect of treatment.

## 5. Conclusions

Our results confirm that *Nosema ceranae* is present throughout Slovakia in all three ecoregions. This is probably related to the gradual displacement of *Nosema apis* by *Nosema ceranae* worldwide. We have confirmed that molecular methods are very sensitive methods for the detection of *Nosema* spp. and have the advantage of being able to determine the species of the pathogen under investigation.

## Figures and Tables

**Table 1:** Shows the numbers of bee queen keepers divide by region in 2021 and 2022.

Number of Bee Queen Breeder		
YEAR	2021	2022
west	23	19
middle	6	5
east	10	8
TOTAL:	39	32

**Table 2:** Microscopical and molecular diagnostic results in 2021 and 2022.

Microscopic Diagnostics in 2021			
Region	number of samples	Positive samples	Negative samples
west	859	182	677
middle	569	142	427
east	600	146	454
TOTAL:	2028	470	1558
**Molecular Diagnostics in 2021**			
Region	number of samples	Positive samples	Negative samples
west	40	34	6
middle	19	18	1
east	22	12	1
TOTAL:	81	73	8
**Microscopic Diagnostics in 2022**			
Region	number of samples	Positive samples	Negative samples
west	915	211	704
middle	568	129	439
east	507	112	395
TOTAL:	1990	452	1538
**Molecular Diagnostics in 2022**			
Region	number of samples	Positive samples	Negative samples
west	195	193	2
middle	123	118	5
east	108	104	4
TOTAL:	426	415	11

**Table 3:** Table of examined values and statistical analyses in %.

Microscopic Diagnostics				
Region	Samples			
	**2021**		**2022**	
	Positive	Negative	Positive	Negative
west	21.19%	78.81%	23.06%	76.94%
middle	24.96%	75.04%	22.71%	77.29%
east	24.33%	75.67%	22.09%	77.91%
Mean	23.49%	76.51%	22.62%	77.38%
Median	24.33%	75.67%	22.71%	77.29%
Min	21.19%	75.04%	22.09%	76.94%
Max	24.96%	78.81%	23.06%	77.91%
R^2^	0.6062	0.6062	0.9745	0.9745
R	0.7785	0.7785	0.9871	0.9871
**Molecular Diagnostics**				
Region	Samples			
	**2021**		**2022**	
	Positive	Negative	Positive	Negative
west	85.00%	15.00%	98.97%	1.03%
middle	94.74%	5.26%	95.93%	4.07%
east	95.45%	4.55%	96.30%	3.70%
Mean	91.73%	8.27%	97.07%	2.93%
Median	94.74%	5.26%	96.30%	3.70%
Minimum Value	85.00%	15.00%	95.93%	1.03%
Maximum Value	95.45%	94.74%	98.97%	4.07%
R^2^	0.8012	0.6504	0.6504	0.8012
R	0.8950	0.8064	0.8064	0.8950

## Data Availability

Not applicable.
